# Diet, lifestyle and gut microbiota composition among Malaysian women with gestational diabetes mellitus: a prospective cohort study

**DOI:** 10.1038/s41598-024-57627-5

**Published:** 2024-03-22

**Authors:** Thubasni Kunasegaran, Vinod R. M. T. Balasubramaniam, Valliammai Jayanthi Thirunavuk Arasoo, Uma Devi Palanisamy, Yen Ker Tan, Amutha Ramadas

**Affiliations:** 1 Jeffrey Cheah School of Medicine and Health Sciences, Monash University Malaysia, 47500 Bandar Sunway, Malaysia; 2 Mackay Base Hospital, Mackay, QLD 4740 Australia

**Keywords:** Diabetes, Risk factors, Applied microbiology

## Abstract

The study addressed a significant gap in the profiling and understanding of the gut microbiota’s influence on Malaysian Malay women with gestational diabetes mellitus (GDM). This prospective cohort study aimed to explore the intricate relationship between gut microbiota, dietary choices, and lifestyle factors among Malay women, both with and without GDM. The research specifically focused on participants during the second (T0) and third (T1) trimesters of pregnancy in Johor Bahru, Malaysia. In Part 1 of the study, a diverse pool of pregnant women at T0 was categorized into two groups: those diagnosed with GDM and those without GDM, with a total sample size of 105 individuals. The assessments encompassed demographic, clinical, lifestyle, and dietary factors at the T0 and T1 trimesters. Part 2 of the study delved into microbiome analysis, targeting a better understanding of the gut microbiota among the participants. Stool samples were randomly collected from 50% of the individuals in each group (GDM and non-GDM) at T0 and T1. The collected samples underwent processing, and 16s rRNA metagenomic analysis was employed to study the microbial composition. The results suggested an association between elevated body weight and glucose levels, poor sleep quality, lack of physical activity, greater intake of iron and meat, and reduced fruit consumption among women with GDM compared to non-GDM groups. The microbiome analysis revealed changes in microbial composition over time, with reduced diversity observed in the GDM group during the third trimester. The genera *Lactiplantibacillus*, *Parvibacter*, *Prevotellaceae UCG001*, and *Vagococcus* positively correlated with physical activity levels in GDM women in the second trimester. Similarly, the genus *Victivallis* exhibited a strong positive correlation with gravida and parity. On the contrary, the genus *Bacteroides* and *Roseburia* showed a negative correlation with omega-3 polyunsaturated fatty acids (PUFAs) in women without GDM in the third trimester. The study highlighted the multifaceted nature of GDM, involving a combination of lifestyle factors, dietary choices, and changes in gut microbiota composition. The findings emphasized the importance of considering these interconnected elements in understanding and managing gestational diabetes among Malaysian Malay women. Further exploration is essential to comprehend the mechanisms underlying this relationship and develop targeted interventions for effective GDM management.

## Introduction

Gestational diabetes mellitus (GDM) is defined as glucose intolerance with the first onset or recognition during pregnancy and is associated with adverse maternal and neonatal outcomes^1,2^. Approximately 1 in 6 live births are affected by diabetes in pregnancy, 84% of which are diagnosed as GDM^1,2^. The global prevalence of GDM has been rising, including in Malaysia, where it contributes to approximately 23.2% of cases^1^. This increase raises significant concerns due to the potential adverse consequences for mothers and their offspring.

Understanding GDM and its root factors is essential for effective prevention and management strategies. Previous research has provided evidence suggesting a potential connection between the gut microbiome and the development and progression of GDM^3–5^. The gut microbiome refers to the community of microorganisms residing in the gastrointestinal tract. It is crucial in various physiological processes, including metabolism and immune function^6^. Furthermore, clinical characteristics, diet intake, lifestyle habits, and alterations in the gut microbial composition have been associated with metabolic disorders and pregnancy-related complications^7,8^.

While a growing body of research explores the relationship between the gut microbiome and GDM, most studies have focused on populations in other regions, and limited research has been conducted on the Malaysian population^9^. The Malaysian government has prioritized research in nutrition, precision medicine, and the promotion of maternal and neonatal health, emphasizing the need to address GDM in the country. Abdullah et al.^9^ found no significant variations in the gut microbiota composition between the first and third trimesters among pregnant women in Malaysia. However, the study lacked detailed dietary and lifestyle information, making it impossible to establish correlations between Malaysian pregnant women's gut microbiota profile, dietary intake, and lifestyle factors. Additionally, the study design did not involve comparing women with medical conditions, such as GDM, with healthy pregnant women.

Given the unique ethnic and cultural context of the Malaysian population, particularly among Malays, who constitute about 70% of women diagnosed with GDM^10^, there is an urgent need for targeted and culturally relevant research. This study addressed existing knowledge gaps by investigating the associations between diet, lifestyle, gut microbiota composition, and GDM among Malaysian Malay women in the second (T0) and third (T1) trimesters.

The research question that guided this study was: How did dietary habits, lifestyle factors, and gut microbiota composition vary between Malaysian Malay women with GDM and those without GDM during the T0 and T1 trimesters? The hypothesis underlying this study posited that specific dietary patterns and lifestyle factors contributed to distinct variations in gut microbiota composition, which, in turn, might have influenced the development and progression of GDM among Malaysian Malay women.

## Methods

### Patient recruitment

This prospective cohort study was conducted in two government clinics, Klinik Kesihatan Tampoi and Klinik Kesihatan Abdul Samad, in Johor Bahru, Malaysia. By performing the study in these government clinics, the research aimed to capture a diverse population of pregnant women from various demographic backgrounds within Johor Bahru. Ethical approval to conduct the study was obtained from the Malaysian Research Ethics Committee (MREC) (NMRR-19-4186-52297) and Monash University Human Research Ethics Committee (MUHREC) (28975). All protocols in the study were performed following relevant guidelines and regulations. Written informed consent was obtained from participants before the recruitment.

The targeted participants were pregnant women of Malay ethnicity with and without GDM in their T0 trimester who had done oral glucose tolerance tests (OGTT). The participants in the study were identified and approached through a collaborative effort with the nurses at the two government clinics. The study objectives and criteria for participation were communicated to the clinic nurses. The involvement of nurses not only facilitated the identification of eligible participants but also helped establish a sense of trust and credibility among the potential participants, increasing the likelihood of their willingness to participate in the study. Both parties identify potential participants based on their medical records and clinical assessments. The records of pregnant women attending the clinics for routine antenatal visits were reviewed to determine their eligibility for the study.

The exclusion criteria were women who are below 18 years and above 45 years old, mixed parentage and non-Malay ethnicity, unable to provide consent due to impairments or severe mental illness as provided in the medical history, diagnosed with other types of diabetes (Type 1, Type 2, Monogenic or secondary) or other morbidities such as gestational hypertension, cardiovascular diseases, gastrointestinal diseases, anemia, and preeclampsia before and during recruitment into the study, having multiple pregnancies and undergoing other therapies or consuming medications (insulin, antibiotics, probiotics, glucose-lowering drugs, or glucose-increasing drugs during recruitment or at any phase after recruitment which could affect the gut microbial profile or study outcomes.

### Sample size calculation

For part 1 of the study, the minimum sample size calculation was done using the web-based EPItools^11^ sample size calculator for the cohort study. The findings by Bowers et al.^12^ reported Asian GDM mothers with high weight gain to have a 4.42 higher likelihood of having a baby born larger than gestational age (LGA). Based on this, a minimum number of 39 participants will be required in each group to give the study 80% power with 95% confidence. Considering possible 20% dropouts due to the study’s prospective design and extreme values, the sample size has been increased to a minimum of 50 pregnant women in each study group (GDM and non-GDM). It is essential to note that the outcome (large gestational age babies) was factored into the calculation as part of objective 4 of the study, which has been excluded in this article.

For Part 2, the sample size for gut microbial analysis was determined based on statistical considerations and feasibility. The minimum sample size for this study was determined using a web-based web application for sample size and power calculation in microbiome studies^13^. Using an operational taxonomic unit (OTU) of 8 as recommended by Fernandez et al.^14^, standard overdispersion of 0.1, and stratification, a minimum sample size of 16 in each group is sufficient to achieve a minimum power of 80%. Additionally, it is prudent to anticipate the possibility of participant dropouts or incomplete data in research studies. By incorporating a slightly larger sample size, this study sought to mitigate the potential impact of such occurrences on the statistical power of study analysis. Therefore, this study opted for a minimum sample size of 25 to guarantee a standard statistical power of at least 80%.

Besides statistical considerations, feasibility was crucial in determining the sample size. Factors such as time constraints, available resources, and the recruitment capacity of the clinics were considered to ensure a realistic and achievable sample size. Hence, for the part 1 study, 105 participants were recruited, with 50 individuals in the GDM group and 55 individuals in the non-GDM group, to ensure an adequate sample size for the overall analysis and to account for potential dropouts or exclusions during the study period. From this sample, 25 participants from each group were randomly selected for stool collection at two different time points (T0 and T1) for the part 2 study. This subset of participants was chosen to obtain prospective data and assess changes in the gut microbial profile over time. The random selection process ensured unbiased representation from both groups and minimized selection bias.

### Data and sample collection

Part 1 of the study recruited pregnant women at T0 and divided them into GDM (n = 50) and non-GDM (n = 55). They completed a questionnaire in the Malay language (Supplementary Table 1) consisting of demographic, clinical, lifestyle, and dietary assessments at the T0 and third trimesters (T1). The dietary intake was assessed using three-day 24-h dietary recalls (2 weekdays and one weekend), and the DietPLUS^®^^15^ was utilized to analyze the energy and nutrient intakes from these recalls. The nutritional data collected from the participants underwent processing to yield energy-adjusted nutrient intakes and detect food groups. This step involved adjusting the nutrient intakes for energy content to account for individual differences in caloric intake. Furthermore, food group analysis was performed to assess the number of servings per day consumed based on different food groups. In addition to the dietary data, the participants' physical activity levels were evaluated using METs (Metabolic Equivalents) calculated from the Global Physical Activity Questionnaire^16^, administered as part of the study instruments. The METs provided a standardized measure of the intensity of physical activity, enabling a comparison of activity levels between the groups. A well-structured questionnaire was used to collect data on participants’ sleeping habits. The questionnaire included specific questions about bedtime and wake-up times during weekdays and weekends, nighttime sleep quality, reasons for waking up during the night, daytime nap duration, and nighttime ambient lighting conditions. Participants rated their nighttime sleep quality on a Likert scale, choosing from “Good,” “Moderate,” or “Not satisfied”. They also selected reasons for waking up during the night from a predefined list. The questionnaire inquired about the duration of daytime naps and the darkness or lightness of the room during nighttime sleep. This structured questionnaire helped collect standardized and detailed information about participants' sleep patterns.

Part 2 of the study involved microbiome analysis. The stool samples were randomly collected from 50% of pool participants from each group: GDM (n = 25) and non-GDM (n = 25). They gave stool samples twice in total, each at T0 and T1. Participants were provided with sterile collection containers (Norgen's Stool Nucleic Acid Collection and Preservation System). Prior to sample collection, participants received a brief explanation of using collection containers. The collected stool samples are frozen at − 80 °C before DNA extraction.

### DNA extraction

Approximately 400 µl of liquid samples were processed using the RNEASY PowerMicrobiome kit from QIAGEN (QIAGEN, Hilden, Germany), following the manufacturer's instructions. The extracted DNA was further assessed for concentration and purity using a SpectraMax QuickDrop Micro-Volume Spectrophotometer from Molecular Devices, USA. The ratio of sample absorbance at 260 and 280 nm was employed to assess DNA purity, with a typical high-quality DNA sample having a 260/280 ratio close to 1.8. The extracted DNA was safely stored at − 20 °C, pending sequencing analysis.

### V3–V4 16s rRNA sequencing: PCR amplification

Barcoded amplicon libraries spanning the V3-V4 hypervariable region of the 16s rRNA gene were prepared using the 341F-805R primer set^17^. Samples were sequenced using the Illumina MiSeq v2 platform, employing the 2 × 250 bp sequencing mode at the Genomics Facility, Monash University Malaysia. Raw sequence reads were subjected to quality control and processing using the DADA2 R package^18^. This package leverages error profiles to precisely define Amplicon Sequence Variants (ASVs). ASVs were subsequently assigned to taxonomy using a pre-trained naïve Bayes classifier trained on the curated 16s rRNA gene database Greengenes v13_8 (99% OTUs). To maintain data quality, ASVs present in fewer than 5% of samples or with fewer than 100 observations and ASVs accounting for less than 0.01% of total reads were filtered from the final dataset before downstream analysis. Sequence depth analyses and rarefaction were also performed using the Phyloseq R package^19^.

### Statistical analyses

All the questionnaire variables were analyzed using IBM SPSS Statistics (version 25). The RStudio software (V.4.2.2) was utilized for gut microbial analysis and its statistical analyses. Initially, the number of reads for each taxon was divided by the total number of reads from all taxa within each sample, enabling the calculation of the relative abundance at different taxonomic levels, including phylum, class, order, family, and genus. The 20 most abundant or frequently observed classes were selected from the data for further analysis.

The relative abundance of a significant class across different conditions was compared using Kruskal–Wallis’s non-parametric test, which is suitable for comparing multiple groups. The resulting p-values were adjusted for multiple comparisons using the False Discovery Rate (FDR) method. Alpha diversity analysis was performed to evaluate the diversity of microbial taxa within each group. Two metrics were used: richness, representing the number of different taxa, and Shannon's index, which accounts for both the richness and evenness of taxa. The cross-sectional difference in alpha diversity between GDM and non-GDM groups at T0 and T1 was assessed using Student's *t*-test.

Additionally, the change in alpha diversity within pregnancy was determined using a repeated measures ANOVA test, comparing GDM status and the different time points. For beta diversity analysis, the significant differences in microbial community composition between GDM and non-GDM women at T0 and T1 were calculated using permutational Multivariate Analysis of Variance (pMANOVA) based on weighted UniFrac distances. The UniFrac distances were determined using the Bray–Curtis distance method.

The “LDA Effect Size (LEfSe)” method was employed to identify specific biomarkers within groups and time points. This method, implemented using the MicrobiomeMarker R package^20^, incorporates the Kruskal–Wallis test, Wilcoxon-Rank Sum test, and Linear Discriminant Analysis (LDA) to generate the LEfSe. Spearman’s rank correlation test was applied to examine the associations between gut microbial profiles and the other variables of interest (clinical characteristics, dietary intake, and lifestyle habits) at the given time points (T0 and T1) in Malaysian Malay women with and without GDM.

## Results

### Part 1: study questionnaires’ findings

This section summarizes the results of the study questionnaires, providing insights into demographic, clinical, lifestyle, and dietary factors among the participants.

### Characteristics of the study population

The demographic and clinical characteristics of the recruited participants are shown in Table 1. The median maternal age for all participants was 31 (IQR = 8), and one-third (66.7%) were between 25 and 34 years. Most pregnant women with at least higher secondary-level education (94.3%) were employed (53.3%). Women with GDM exhibited significantly higher fasting blood glucose (FBG) and 2-h postprandial glucose (2-HPP) compared to the women without GDM (p < 0.001, respectively). Women with GDM also experienced higher weight gain at T0 (p = 0.031) with a median weight of 4 (IQR = 4.8) and 6 (IQR = 3.0) at T1 (p = 0.044) compared to women without GDM. Lastly, the mean blood pressure was higher among women with GDM.

**Table 1:** Demographic and clinical characteristics of study participants at recruitment (N = 105).

		Total (N = 105)	Non-GDM (n = 55)	GDM (n = 50)	P-value
Maternal age (years)	Median (IQR)	31 (8.0)	30.0 (8.0)	32.5 (7.0)	0.139
	18–24	9 (8.6)	5 (9.1)	4 (80.0)	0.762
	25–34	70 (66.7)	38 (69.1)	32 (64.0)	
	35–45	26 (24.8)	12 (21.8)	14 (28.0)	
Education	Primary	1 (1)	0 (0)	1 (2.0)	0.156
	Lower Secondary	5 (4.8)	1 (1.8)	4 (8.0)	
	Higher Secondary	52 (49.5)	25 (45.4)	27 (54.0)	
	Tertiary	47 (44.8)	29 (52.7)	18 (36.0)	
Occupation	Housewife	49 (46.7)	25 (45.4)	24 (48.0)	0.326
	Employed	56 (53.3)	30 (54.5)	26 (52.0)	
Household income (MYR)	< 1000	1 (1)	1 (1.8)	0 (0)	0.030*
	1000–1999	17 (16.2)	11 (20.0)	6 (12.0)	
	2000–2999	22 (21.0)	7 (12.7)	15 (30.0)	
	3000–3999	22 (21.0)	8 (14.5)	14 (28.1)	
	> 4000	43 (41.0)	28 (50.9)	15 (30.0)	
History of GDM	Yes	17 (16.2)	6 (10.8)	11 (22.0)	0.184
	No	88 (83.8)	49 (89.1)	39 (78.0)	
Family history of diabetes	Yes	45 (42.9)	21 (38.2)	24 (48.0)	0.310
	No	60 (57.1)	34 (61.8)	26 (52.0)	
FBG (mmol/L)	Median (IQR)	4.2 (0.8)	3.9 (0.6)	4.5 (0.78)	< 0.001**
2-HPP (mmol/L)	Median (IQR)	7.1 (2.7)	5.5 (1.5)	8.4 (0.95)	< 0.001**
Gravida	Median (IQR)	2 (2.0)	2 (2.0)	2 (3.0)	0.458
Parity	Median (IQR)	1 (2.0)	1(2.0)	1(3.0)	0.330
Pre-pregnancy body weight (kg)	Median (IQR)	62 (20.8)	58 (22.0)	64 (23.3)	0.150
Pre-pregnancy BMI (kg/m^2^)	< 18.5	10 (9.5)	6 (10.9)	4 (8.0)	0.215
	18.5–24.9	39 (37.1)	25 (45.5)	14 (28.0)	
	25.0–29.9	29 (27.6)	12 (21.8)	17 (34.0)	
	> 30.0	27 (25.7)	12 (21.8)	15 (30.0)	
Weight gain (kg)					
Second trimester	Median (IQR)	3 (4.4)	2 (4.0)	4 (4.8)	0.031*
Third trimester	Median (IQR)	6 (3.0)	5 (3.0)	6 (3.0)	0.044*
Systolic BP (mmHg)					
Second trimester		111.5 (16)	110 (17)	112.0 (13)	0.170
Third trimester		117.0 (15)	111 (18)	119 (18)	0.042*
Diastolic BP (mmHg)		70.5 (12)	68 (11)	73 (10)	0.006*
Second trimester		78 (14)	76 (11)	79 (18)	0.086
Third trimester					

*GDM* gestational diabetes mellitus, *MYR* ringgit Malaysia (1MYR = 0.214USD); n (%) based within groups. *Significant at p < 0.05; **significant at p < 0.001; n (%) based within groups; *IQR* interquartile range, *GDM* gestational diabetes mellitus, *FBG* fasting blood glucose, *2-HPP* 2 h postprandial, *BMI* body mass index, *BP* blood pressure. Significant values are in bold.

### Comparison of lifestyle and dietary factors between study groups at second and third trimesters

Table 2 compares lifestyle factors between the study groups at T0 and T1. A higher proportion of the women with GDM (22.5%) were not satisfied with their sleep quality compared to pregnant women without GDM at T1. Conversely, the darkness level during sleep was associated with the study groups only at T0 (p = 0.018). The level of physical activity was associated with the distribution of the participants according to study groups at T0 but not at T1. The proportion of women with GDM with high physical activity at T0 appears lower than those without GDM (p = 0.035).

**Table 2:** Lifestyle characteristics of study participants in second (N = 105) and third (N = 84) trimesters.

		Second trimester (T0)			Third trimester (T1)		
		Non-GDM (n = 55)	GDM (n = 50)	P-value	Non-GDM (n = 44)	GDM (n = 40)	P-value
Smoking							
History of smoking	Passive-smoker	13 (23.6)	21(42.0)	0.060	10 (22.7)	15 (37.5)	0.107
	Non-smoker	42 (75.4)	29 (58.0)		34 (77.3)	25 (62.5)	
Sleeping habits							
Sleeping quality	Satisfied	28 (50.9)	21 (42.9)	0.410	6 (13.6)	2 (5.0)	0.030*
	Moderate	27 (49.1)	28 (57.1)		36 (81.8)	29 (72.5)	
	Not satisfied	0 (0.0)	1 (2.0)		2 (4.5)	9 (22.5)	
Darkness during sleep	Unable to read but able to view the room	4 (7.3)	6 (12.0)	0.018*	6 (13.7)	4 (10.0)	0.395
	Unable to view the room but able to view extremities	35 (63.6)	18 (36.0)		29 (65.9)	22 (55.0)	
	Complete darkness	16 (29.1)	26 (52.0)		9 (20.5)	14 (35.0)	
Duration of sleeping (hours/day)	Weekday, Median (IQR)	8 (2)	7 (2.0)	0.056	8 (2)	7 (3)	0.336
	Weekend, Median (IQR)	8 (2)	8 (1.6)	0.861	8 (3)	9 (3)	0.052
	Nocturnal sleep, Median (IQR)	1 (1)	1 (0.7)	0.600	1 (1)	2 (4)	0.203
Physical activity							
Physical activity level	Low	26 (47.3)	20 (40.6)	0.035*	24(54.5)	21(52.5)	0.056
	Moderate	11 (20.0)	21 (42.0)		11(25.0)	17 (42.5)	
	High	18 (32.7)	9 (18.0)		9 (36.4)	2 (5.0)	
Sedentary behavior	≥ 8 h sitting time/day	21 (38.2)	23 (46.0)	0.436	21 (47.7)	23 (57.5)	0.391
	< 8 h sitting time/day	34 (61.8)	27 (54.0)		23 (52.3)	17 (42.5)	

*Significant at p < 0.05; n (%) based within groups; *SD* standard deviation, *IQR* interquartile range, *GDM* gestational diabetes mellitus, *MET* metabolic equivalents. Significant values are in bold.

Table 3 compares the dietary intake between T0 and T1 trimesters study groups. Interestingly, the GDM group reported a lower median intake of carbohydrates (129.1 g/1000 kcal vs 139.2 g/1000 kcal, p = 0.005) and total fats (7.5 g/1000 kcal vs 41.9 g/1000 kcal, p = 0.001) compared to the non-GDM group at T1. No differences can be observed in any macronutrient intake in T0.

**Table 3:** Dietary intake of study participants in second (N = 105) and third (N = 84) trimesters.

	Second trimester (T0)			Third trimester (T1)		
	Non-GDM (n = 55)	GDM (n = 50)	P-value	Non-GDM (n = 44)	GDM (n = 40)	P-value
Macronutrient intake (g/1000 kcal), median (IQR)						
Carbohydrates	126.3 (26.4)	127.2 (21.0)	0.734	139.2 (21.3)	129.1 (20.5)	0.005*
Protein	38.17 (9.4)	37.7 (7.4)	0.302	41.9 (8.3)	41.0 (7.2)	0.802
Total fats	36.9 (9.1)	38.7 (7.7)	0.599	30.1 (8.3)	7.5 (8.1)	0.001**
Micronutrient intake (mg/1000 kcal), median (IQR)						
Vitamin B1	0.44 (0.2)	0.4 (0.1)	0.404	0.54 (03)	0.5 (0.3)	0.441
Vitamin B2	0.7 (0.3)	0.7 (0.2)	0.862	0.7 (0.4)	0.7 (0.2)	0.490
Vitamin B3	6.6 (2.8)	6.6 (1.3)	0.621	6.6 (2.6)	6.3 (2.5)	0.816
Vitamin C	33.7 (26.7)	25.8 (22.0)	0.546	43.5 (48.3)	29.6 (25.4)	0.009*
Calcium	283.8 (112.3)	258.7 (162.4)	0.724	276.0 (139.4)	279.0 (175.4)	0.707
Iron	6.8 (1.8)	8.1 (3.4)	0.012*	8.4 (2.7)	7.9 (2.3)	0.295
Phosphorus	513.1 (204.5)	531.1 (204.5)	0.401	557.4 (180.1)	596.8 (151.2)	0.055
Potassium	930.0 (471.4)	968.9 (432.9)	0.837	769.1 (471.4)	602.7 (558.2)	0.055
Retinol^a^	343.4 (449.1)	538.5 (721.8)	0.033*	371.5 (296.7)	308.2 (208.2)	0.567
Sodium	1582.9 (734.7)	1524.4 (675.3)	0.691	1736.0 (564.3)	1834.8 (749.1)	0.687
Other non-nutrients (g/1000 kcal), median (IQR)						
Dietary fiber	8.0 (3.4)	7.3 (2.6)	0.695	7.9 (3.2)	8.4 (2.6)	0.747
Cholesterol^b^	124.2 (68.2)	125.7 (42.6)	0.939	125.0 (72.2)	154.2 (86.8)	0.032*
Sugar	32.4 (14.3)	31.5 (14.2)	0.434	35.2 (19.4)	31.9 (15.0)	0.036*
Omega 6 PUFA	4.3 (1.9)	4.5 (1.2)	0.461	3.9 (1.2)	4.5 (2.0)	0.021*
Omega 3 PUFA	0.2 (0.1)	0.18 (0.1)	0.116	0.1 (0.1)	0.2 (0.1)	0.223
Food groups, median servings per day (IQR)						
Vegetables	0.7 (1.2)	1.1 (1.7)	0.034*	1.0 (1.2)	1.2 (1.0)	0.674
Fruits	0.5 (1.0)	0.5 (0.6)	0.562	0.4 (1.3)	0.1 (0.3)	0.013*
Meat	1.1 (1.5)	2.3 (2.3)	< 0.001**	1.3 (1.7)	1.8 (1.5)	0.258
Fish and seafood	0.6 (1.7)	0.7 (1.2)	0.64	0.3 (0.7)	0.4 (0.4)	0.224
Dairy products	12.0 (5.1)	15.3 (5.0)	< 0.001**	9.0 (4.4)	12.7 (5.0)	0.001**
Fats and oils	1.3 (2.9)	1.5 (3.0)	0.196	0.1 (2.3)	0.3 (2.8)	0.835

^a^Measured as RE; ^b^Measured as mg/1000 kcal; *Significant at p < 0.05; **significant at p < 0.001; n (%) based within groups; *IQR* interquartile range, *GDM* gestational diabetes mellitus. Significant values are in bold.

Women with GDM exhibited significantly higher median intake of iron (8.1 mg/1000 kcal vs 6.8 g/1000 kcal, p = 0.012) and retinol (538.5 mg/1000 kcal vs 343.4 mg/1000 kcal, p = 0.033) compared to women without GDM at T0. However, such differences were not seen at T1. Median vitamin C intake was reported to be significantly lower in the GDM group compared to the non-GDM group at T1 (29.6 mg/1000 kcal vs 43.5 mg/1000 kcal, p = 0.009). While the median sugar intake was significantly lower in the GDM group, the median cholesterol and omega 3 PUFA intake was higher.

The median intake of vegetables (1.1 servings/day vs. 0.7 servings/day, p = 0.034), meats (2.3 servings/day vs 1.1 servings/day, p < 0.001), and fats and oils (15.3 servings/day vs 12.0 servings/day, p < 0.001) were significantly higher in the GDM group, compared with the non- GDM group at T0. While the overall fats and oils intake was reduced in T1, the consumption was still relatively higher in the GDM group (p < 0.001). However, the median intake of fruits was lower in the GDM group than in the non-GDM group at T1 (0.1 servings/day vs 0.4 servings/day, p = 0.013).

### Part 2: microbiome analysis findings

This section presents microbiome analysis results, highlighting changes in microbial composition over time and any significant correlations with the participants' characteristics, such as dietary habits and lifestyle factors.

### Comparison of alpha diversity of gut microbiota between groups in the second and third trimesters

**Figure 1:**
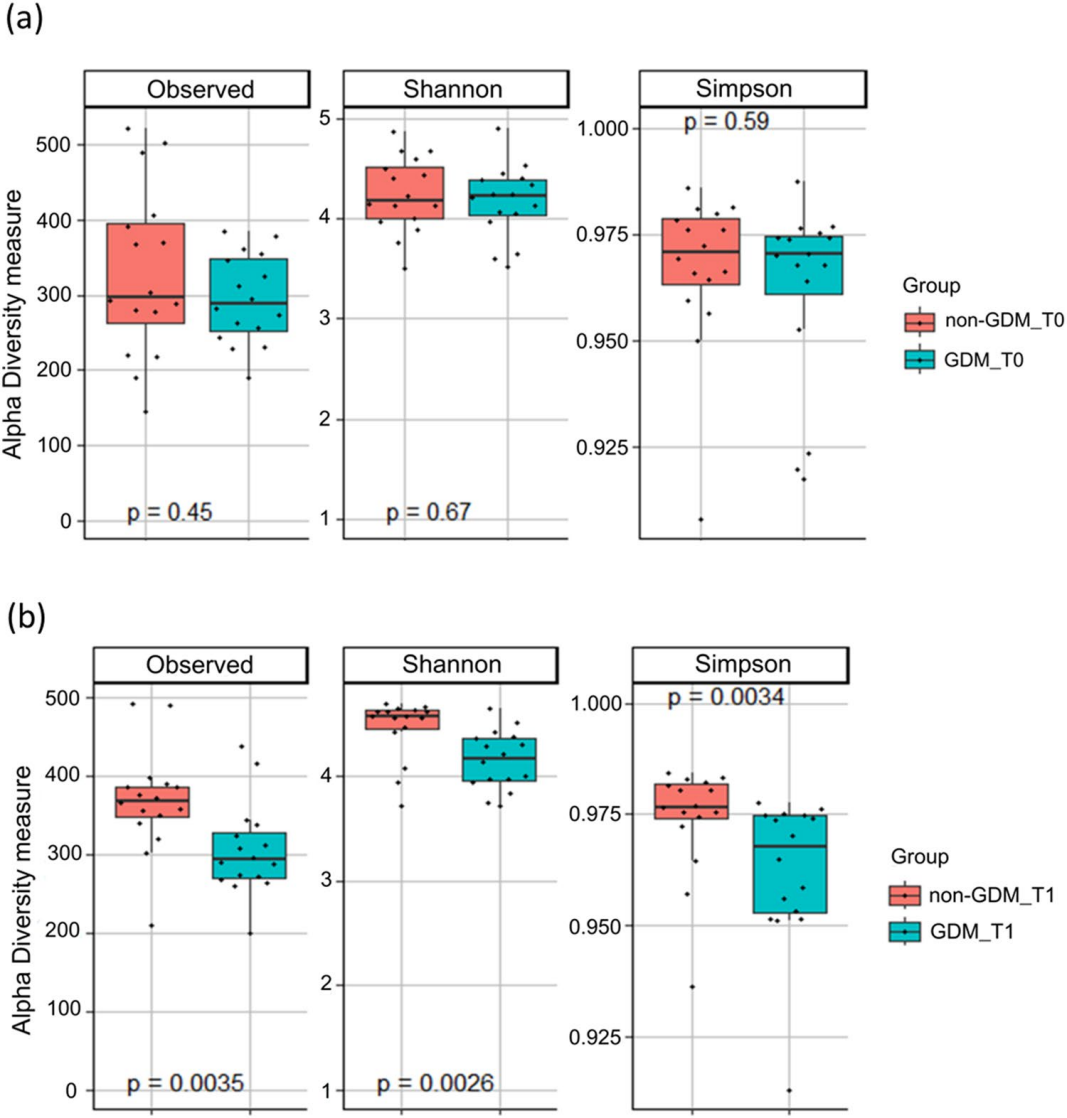
Alpha diversity measures for non-GDM and GDM groups in the second trimester (T0) (**a**) and in the third trimester (T1) (**b**).

The findings indicated no significant differences in alpha diversity at T0 between groups (Fig. 1a). In contrast, notable differences were observed at T1 between pregnant women with gestational diabetes mellitus (GDM) and those without GDM (Fig. 1b). The beta diversity analysis also demonstrated relatively similar microbial community compositions between the non-GDM and GDM groups at T0 and T1 (data not shown).

### Taxonomic analysis of microbial communities at the genus level for GDM and non-GDM groups at second and third trimesters

**Figure 2:**
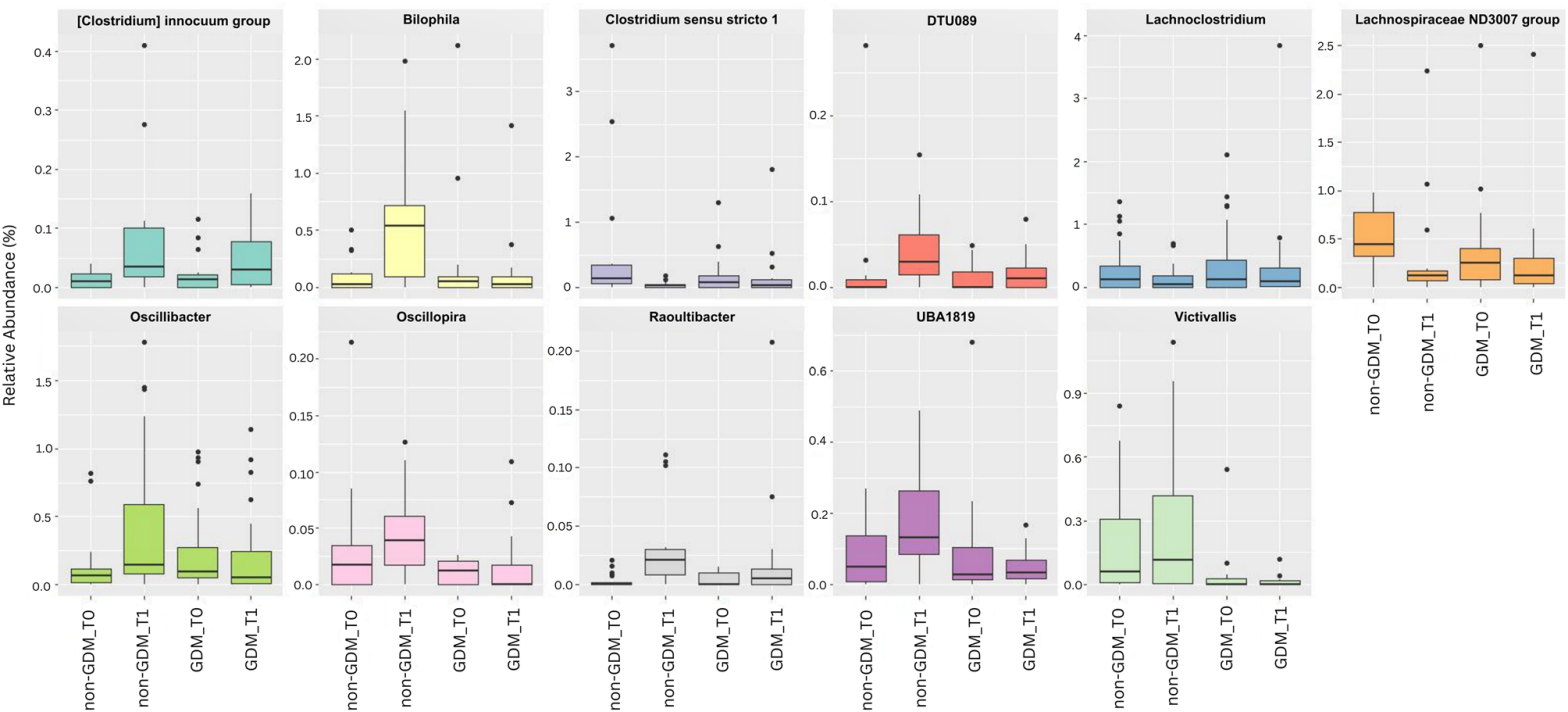
Taxonomic analysis of microbial communities at the genus level.

The taxonomic analysis conducted at the genus level revealed significant differences in the relative abundance of specific microbial genera among different groups (Fig. 2). Firstly, the abundance of *Bilophila* showed a significant difference between the non-GDM group in the T0 and T1 trimesters. It suggests that the relative abundance of *Bilophila* may change as pregnancy progresses. Similarly, the *Lachnospiraceae ND3007* group exhibited a significant difference (p = 0.024) in abundance between the non-GDM group at T0 and T1 (Table 4), indicating that this specific group of bacteria may undergo compositional changes during pregnancy. Furthermore, the abundance of *Oscillibacter* showed a significant difference (p = 0.003) between the non-GDM group at T0 and T1, suggesting that the progression of pregnancy may influence the relative abundance of *Oscillibacter*.

**Table 4:** Comparison of genus-level gut microbial profiles between non-GDM and GDM groups at second and third trimesters.

Genus	Group 1	Group 2	Adjusted p-value
*Bilophila*	Non-GDM_T0	Non-GDM_T1	0.024*
*Lachnospiraceae ND3007* group	Non-GDM_T0	Non-GDM_T1	0.042*
*Lachnospiraceae ND3007* group	Non-GDM_T0	GDM_T0	0.037*
*Oscillibacter*	Non-GDM_T0	Non-GDM_T1	0.003**
*Victivallis*	Non-GDM_T1	GDM _T1	0.009*
*[Clostridium] innocuum* group	Non-GDM_T0	Non-GDM_T1	0.027*

*Significant at p < 0.05; **significant at p < 0.005; *T0* second trimester, *T1* third trimester.

Additionally, the abundance of *Victivallis* displayed a significant difference (p = 0.009) between the non-GDM group at T1 and the GDM group at T1. It suggests a potential association between the presence or abundance of *Victivallis* and GDM during the mid-third trimester. Lastly, the *[Clostridium] innocuum* group exhibited a significant difference (p = 0.027) in abundance between the non-GDM group at T0 and T1, indicating that the relative abundance of this group may also change as pregnancy progresses.

### Identification of marker abundance

**Figure 3:**
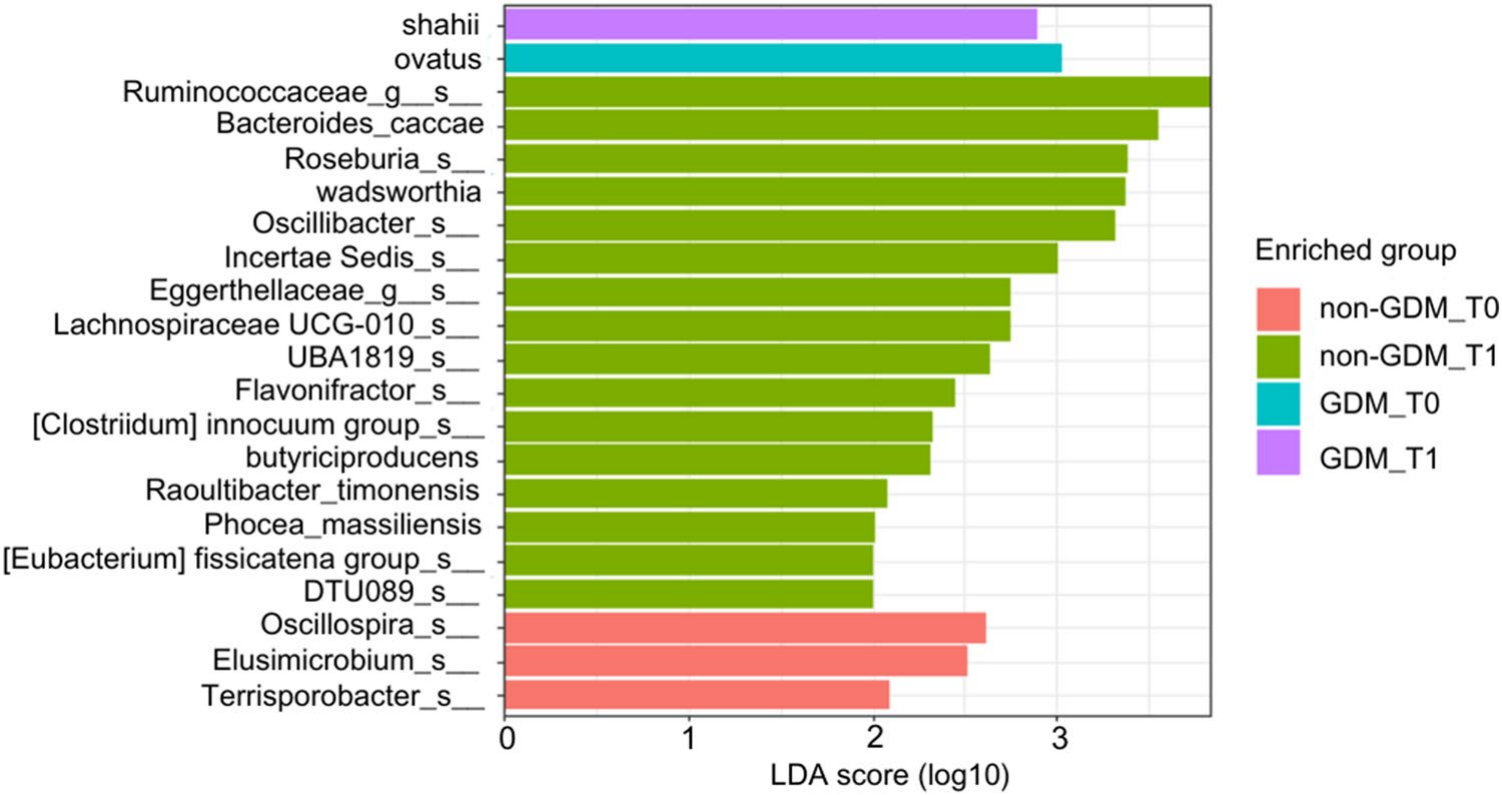
Bar plot showing the LDA score (log 10) for the abundance of potential biomarker taxa identified using the LEfSe approach for the two groups (non-GDM and GDM) at T0 and T1.

In the non-GDM group at T0, three microbial taxa, species *Oscillospira*, *Elusimicrobium*, and *Terrisporobacter*, showed enrichment compared to other groups (Fig. 3). In the non-GDM group at T1, several microbial taxa, including genus and species *Ruminococcaceae*, *Bacteroides_caccae*, *Eggerthellaceae*, *Incertae Sedis,* and species *Roseburia*, *wadsworthia*, *Oscillibacter*, *Lachnospiraceae UCG010*, *UBA1819*, *Flavonifractor*, *[Clostridium] innocuum group*, *butyriciproducens*, *Raoultibacter_timonensis*, *Phocea_massiliensis*, *[Eubacterium] fissicatena group*, *DTU089*, exhibited enrichment compared to other groups.

In the GDM group at T0, the species *Ovatus* showed enrichment to other groups. In the GDM group at T1, the species *Shahii* belonging to the genus *Alistipes* showed enrichment compared to other groups. Figure 3 presents the bar plot for the Linear Discriminant Analysis (LDA) score (log 10). Supplementary Table 2 provides information on the enrichment of specific microbial features in two groups (non-GDM and GDM at T0 and T1) and their corresponding effect sizes linear discriminant analysis (ef_lda), p-values, and FDR.

### Correlation between demographic, clinical, lifestyle, and nutrient intake factors to genus taxonomic rank for GDM and non-GDM groups in the second and third trimesters

**Figure 4:**
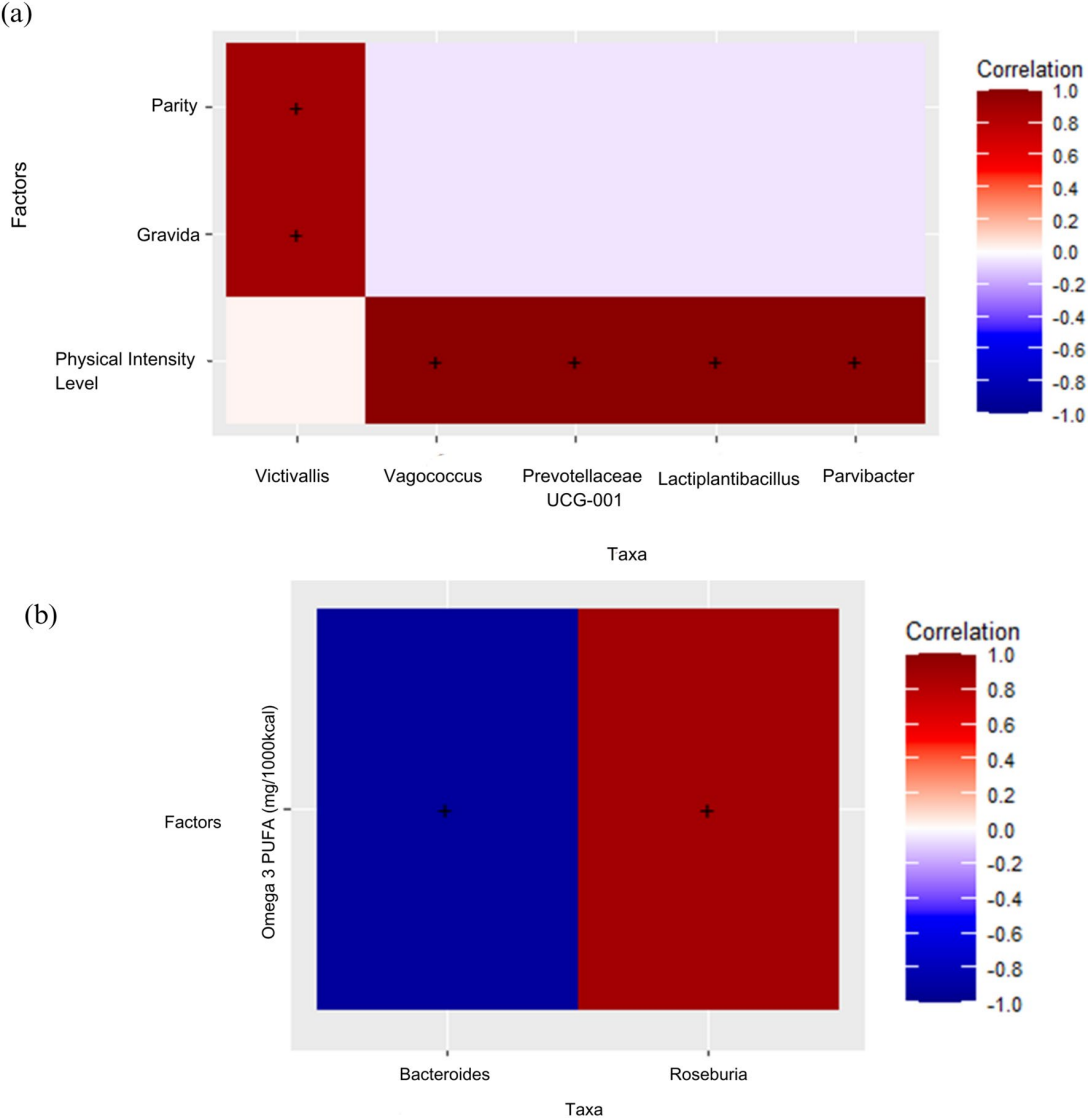
Correlation plot showing Spearman’s correlation coefficient values between gut microbial taxa and lifestyle and clinical factors in the GDM group in the second trimester (T0) (**a**) and correlation plot between gut microbial taxa and Omega-3 PUFA intake (mg/1000 kcal) in the non-GDM group in the third trimester (T0) (**b**). (**a**) Red color represents stronger positive correlations. The X-axis represents the microbial taxa, and the Y-axis represents the parity, gravida, and physical intensity level. ‘+’ signs represent observations with a significant correlation at p-value < 0.05. (**b**) Darker colors represent stronger positive correlations. The X-axis represents the microbial taxa, and the Y-axis represents the nutrient factor. ‘+’ signs represent observations with a significant correlation at p-value < 0.05. Omega3 PUFA (mg/1000 kcal) refers to omega-3 polyunsaturated fatty acids intake per 1000 kcal.

Several significant correlations were observed in the correlation analysis between clinical, lifestyle, and nutrient intake factors and the genus-level taxonomic rank for the GDM group in the T0. Specifically, four genera, namely *Lactiplantibacillus*, *Parvibacter*, *Prevotellaceae UCG001*, and *Vagococcus*, positively correlated with physical activity level (Supplementary Table 3). It indicates that a higher level of physical intensity was associated with an increased abundance or presence of these genera in the microbial communities of the GDM group at T0. Additionally, the genus *Victivallis* exhibited a strong positive correlation with gravida (number of pregnancies) and parity (number of previous live births). These correlations suggest that a higher number of pregnancies and previous live births were associated with an increased abundance or presence of *Victivallis* in the microbial communities of the GDM group at T0. These findings highlight potential associations between specific clinical and lifestyle factors and the composition of microbial communities in individuals with GDM at T0. Figure 4a represents the plot of Spearman’s correlation coefficient values between gut microbial taxa and lifestyle and clinical factors in the GDM group at T0. Supplementary Table 3 presents the detected significant correlation values for the GDM group at T0. Conversely, no significant correlations were observed in the analysis of the correlation between clinical, lifestyle, and nutrient intake factors and genus taxonomic rank for the non-GDM group at T0 (results not shown).

The findings were contradictory in the T1 (Fig. 4b). No significant correlations were found in the correlation analysis between clinical, lifestyle, and nutrient intake factors and the genus-level taxonomic rank for the GDM group at T1. It suggests no strong associations between these factors and the abundance or presence of specific genera in the microbial communities of individuals with GDM at T1. However, there are two significant correlations for the non-GDM group at T1.

First, the genus *Bacteroides* showed a strong negative correlation with omega-3 polyunsaturated fatty acids (PUFAs). It suggests that higher levels of omega-3 PUFA intake were associated with decreased abundance or presence of *Bacteroides* in the microbial communities of the non-GDM group at T1 (Supplementary Table 4). *Bacteroides* is a commonly found genus in the gut microbiota and plays a role in various metabolic processes.

On the other hand, the genus *Roseburia* exhibited a strong positive correlation with omega-3 PUFAs. It indicates that higher levels of omega-3 PUFA intake were associated with increased abundance or presence of *Roseburia* in the microbial communities of the non-GDM group at T1. *Roseburia* is known for its ability to produce short-chain fatty acids, which benefit gut health. These findings suggest a potential link between the intake of omega-3 PUFAs and the composition of microbial communities in individuals without GDM at T1. Figure 4b represents the plot of Spearman’s correlation coefficient values between gut microbial taxa and lifestyle and clinical factors in the non-GDM group at T1. Supplementary Table 4 presents the detected significant correlation values for the non-GDM group at T1.

## Discussion

In addressing the study's first objective, we sought to discern differences in clinical factors, dietary intake, and lifestyle habits between Malaysian Malay women with and without GDM at the T0 and T1 trimesters. The investigation revealed distinct patterns between the two groups at both time points. Participants with GDM exhibited elevated FBG levels, 2-HPP levels, and increased body weight. Furthermore, they were more likely to fall into the overweight and obese BMI categories. Notable variations in lifestyle habits, including sleep quality, darkness during sleep, and physical activity levels, were observed. Dietary intake also differed, with variations in iron, meat, fats/oils, carbohydrates, and fruit consumption.

In comparison, the current study aligns with systematic reviews^21,22^ that have consistently reported higher FBG and postprandial levels in GDM groups, indicating poorer glucose tolerance. Notable changes in weight and BMI categories were observed among Malaysian Malay women with and without GDM in the T1, compared to the earlier T0 stage. The higher weight and increased likelihood of being overweight or obese in the GDM group align with previous studies that have reported an association between GDM and higher BMI^23–25^. Changes in weight and BMI during pregnancy, particularly in the context of GDM, require careful consideration. It is expected that women will experience weight gain during pregnancy due to factors such as fetal growth, increased blood volume, and expansion of maternal tissues^26^. Monitoring weight gain and ensuring it aligns with recommended guidelines based on pre-pregnancy BMI is crucial.

Women with GDM had higher total fat intake than those without GDM at T0. This higher fat intake could contribute to increased insulin resistance and impaired glucose metabolism, factors associated with GDM development^27,28^. Additionally, the higher iron intake observed in the GDM group aligns with systematic reviews reporting higher iron requirements during pregnancy, particularly in women with GDM^29^. Iron is crucial for maintaining hemoglobin levels and preventing iron deficiency anemia, which can negatively affect maternal and fetal health^30–32^. However, it is essential to note that pregnant women, especially those with other known GDM risk factors, should avoid excessive heme iron-enriched food^29^. Further research is needed to explore the relationship between iron-enriched foods and GDM within the Malaysian Malay population.

Moving to the T1 trimester, the GDM group showed increased meat and fats/oils consumption compared to the non-GDM group. It aligns with studies suggesting a potential association between GDM and a higher intake of animal-based foods and unhealthy fats^33,34^. While lean meat sources can provide essential nutrients, excessive red or processed meat intake may increase saturated fat and cholesterol levels, potentially exacerbating insulin resistance^28,35^. Moreover, the higher intake of fat/oils in the GDM group could contribute to excessive calorie intake and impact glycemic control^36^. Carbohydrate intake, another crucial aspect in GDM management, exhibited variations between the two groups. The GDM group had higher carbohydrate intake at T0, highlighting the importance of monitoring carbohydrate consumption to prevent rapid blood glucose spikes and challenges in glycemic control^37,38^. At T1, both groups showed a decrease in carbohydrate intake, reflecting dietary modifications aimed at managing GDM through carbohydrate monitoring and control. Fruit consumption, often recommended for its valuable nutrient content and fiber, was lower in the GDM group at T0 and T1. Furthermore, lower fruit consumption may be associated with limited nutrient diversity and inadequate fiber intake^39,40^, potentially impacting GDM management. This finding raises concerns as fruits provide essential vitamins, minerals, and dietary fiber that support overall health and glycemic control.

The lower satisfaction with sleep quality reported by the GDM group aligns with previous research suggesting a relationship between poor sleep quality and an increased risk of GDM^41^. Disrupted sleep patterns and inadequate sleep duration have been linked to insulin resistance and impaired glucose metabolism, critical factors in GDM pathogenesis^42,43^. Interventions aimed at improving sleep quality and promoting healthy sleep habits may have implications for preventing and managing GDM. The difference in darkness during sleep is an intriguing finding. The higher proportion of participants in the GDM group reporting complete darkness during sleep may indicate an association between melatonin secretion and GDM. Melatonin, a hormone released during the night, has been shown to play a role in glucose homeostasis and insulin sensitivity^44,45^. Further research is needed to understand the underlying mechanisms and potential interventions related to light exposure during sleep in the context of GDM.

Physical activity levels also differed between the two groups, with the GDM group having a lower proportion of individuals with high physical activity levels. This finding is consistent with a systematic review suggesting an inverse relationship between higher physical activity and GDM risk^46^. Regular physical activity has improved insulin sensitivity, glucose metabolism, and cardiovascular health^47,48^. Encouraging pregnant women, including those with GDM, to engage in appropriate physical activities under healthcare guidance can improve glycemic control and overall health outcomes.

The study revealed noteworthy insights in addressing the second objective centered on discerning differences in gut microbial profiles between Malaysian Malay women with and without GDM at the T0 and T1 trimesters. The analysis of gut microbial profiles revealed significant disparities in the abundance of specific microbial groups at different taxonomic levels between the non-GDM and GDM groups. Surprisingly, at T0, no substantial differences in microbial diversity emerged between the two groups. However, by T1, the GDM group demonstrated a marked reduction in alpha diversity compared to the non-GDM group. These outcomes substantiate the initial hypotheses, implying that GDM may influence the composition of the gut microbiome. The unexpected absence of diversity differences at T0 and the subsequent decline in alpha diversity at T1 underscore the dynamic nature of the gut microbiome during pregnancy, with the impact of GDM becoming more apparent in later stages.

Furthermore, the present findings are consistent with previous research suggesting significant differences in the gut microbial diversity and composition between women with and without GDM during different stages of pregnancy. For instance, Koren et al. conducted a study on 91 pregnant women with varying pre-pregnancy BMI and gestational diabetes status and their infants to understand their role in pregnancy better^49^. They found that the gut microbiota underwent significant changes from the first to the third trimester of pregnancy. These changes included a remarkable expansion of microbial diversity among mothers, an overall increase in the abundance of Proteobacteria and Actinobacteria, and a reduction in overall microbial richness. These findings highlight the dynamic nature of the gut microbiota during pregnancy and suggest that the third trimester is a critical period of microbial community remodeling. Similarly, Crusell et al. examined the gut microbiota of pregnant women with (n = 50) and without GDM (n = 157) and found significant differences in microbial diversity and abundance^3^ and observed distinct microbial profiles associated with GDM. The diagnosis of GDM during the third trimester of pregnancy is associated with an altered composition of the gut microbiota compared to pregnant women with normal blood sugar levels^3^. The present study and previous investigations^3,49,50^ provide compelling evidence for the influence of GDM on gut microbiome composition. It suggests that GDM, as a metabolic disorder, can impact the diversity and abundance of microbial taxa in the gut as pregnancy progresses. It supports the concept that metabolic dysregulation associated with GDM may extend beyond glucose metabolism and affect other physiological processes, including the gut microbiota.

Furthermore, in the present study, the taxonomic analysis at the genus level revealed a difference in the abundance of *Victivallis* between the non-GDM and GDM groups. It is significantly reduced in the GDM group than non-GDM at T1. This finding suggests a potential association between the reduced abundance of *Victivallis* and the development of GDM. However, there is a lack of existing literature specifically exploring the abundance and activities of *Victivallis* in disease development. *Victivallis* is a recently described genus, and its role in the gut microbiome, diseases, and complete characterization is yet to be fully understood^51,52^. Further investigations are necessary for the species *Victivallis* due to its recent discovery and limited understanding of its role in the gut microbiome and its potential implications for diseases such as GDM. Understanding the functional significance of *Victivallis* in GDM pathophysiology is crucial to gain insights into its potential involvement in metabolic regulation, inflammation, and other relevant processes. Identifying reduced *Victivallis* abundance in Malaysian Malay pregnant women with GDM compared to non-GDM during the third trimester highlights the need for further research to validate and expand upon these findings.

The analysis provided valuable insights in exploring the final objective to reveal associations between clinical factors, dietary intake, lifestyle habits, and gut microbial profiles during pregnancy (T0 and T1) in Malaysian Malay women with and without GDM. Positive correlations were observed between *Bacteroides* and omega-3 PUFAs in Malaysian Malay pregnant women without GDM at T1. Negative correlations were observed between *Roseburia* and omega-3 PUFAs. It suggests a potential link between dietary intake of omega-3 PUFAs and gut microbiota composition during the later stages of pregnancy. Omega-3 PUFAs are known for their anti-inflammatory properties and have been associated with various health benefits. Previous studies have also reported associations between dietary factors, particularly omega-3 PUFAs, and gut microbiota composition^53–57^. However, further research is needed to understand the underlying mechanisms and their implications for maternal and fetal health.

The study also identified positive correlations between specific microbial taxa (*Lactiplantibacillus*, *Parvibacter*, *Prevotellaceae UCG001*, *Vagococcus*, and *Victivallis*) and physical activity levels in Malaysian Malay pregnant women with GDM at T0. Physical activity has been recognized as a modulator of the gut microbiome, influencing microbial diversity and composition. The findings suggest that physical activity level during the T0 may be associated with specific microbial taxa in GDM individuals, highlighting the potential impact of exercise on the gut microbiome in the context of GDM^58–60^.

Furthermore, correlations were observed between microbial taxa and gravida and parity. Gravida refers to the number of pregnancies a woman has had, while parity refers to the number of births. Previous research has shown that parity influences the gut microbiota of offspring^61^, indicating its impact beyond the mother. These associations suggest that the reproductive history of GDM individuals, as indicated by gravida and parity, may contribute to variations in the gut microbial composition during the T0 among Malaysian Malay women.

Indeed, the observed microbial variations in Malaysian Malay women with and without GDM raise exciting questions about the potential role of gut microbiota in developing and progressing GDM. While the exact mechanisms are still being investigated, several hypotheses can be proposed based on the current findings.

Firstly, the gut microbiota has been implicated in regulating glucose metabolism and insulin sensitivity^62,63^. Disruptions in the composition and diversity of gut microbiota in Malaysian Malay pregnant women with GDM may lead to impaired glucose metabolism and insulin resistance, both critical factors in the development of GDM.

Secondly, imbalances in gut microbiota can trigger an inflammatory response and affect immune regulation^64,65^. Chronic low-grade inflammation contributes to insulin resistance and the pathogenesis of GDM. Changes in the gut microbial composition in Malaysian Malay pregnant women with GDM may influence the production of inflammatory markers and modulate the immune response, thereby influencing the risk of GDM.

Thirdly, the current study's findings suggest that specific bacterial markers associated with butyrate production exist in non-GDM individuals but not those with GDM. These markers include taxa such as *Ruminococcaceae*, *Roseburia* species, *Oscillibacter* species, *Lachnospiraceae UCG010*, *Flavonifractor,* *Butyriciproducens*, *[Clostridium] innocuum group*, and *Bacteroides_caccae*. Butyrate, a short-chain fatty acid (SCFA), is produced by certain bacteria in the gut through the fermentation of dietary fibers^66,67^. It has been recognized as an essential energy source for colonocytes and has shown the ability to regulate glucose and lipid metabolism^68^. The correlation between butyrate-producing bacteria and metabolic homeostasis suggests that these bacteria may be crucial in maintaining metabolic health. The current findings imply that a lack of abundance of butyrate-producing bacteria, as observed in GDM individuals, may have implications for glucose and lipid metabolism regulation. Consequently, alterations in the gut microbiota, precisely the absence or reduced abundance of specific butyrate-producing bacteria in the GDM group, may contribute to the development or progression of GDM.

Lastly, dysbiosis in the gut microbiota can increase gut permeability, allowing the translocation of bacterial products such as lipopolysaccharides (LPS) into the bloodstream^69,70^. This phenomenon, called endotoxemia, may trigger an inflammatory response and contribute to insulin resistance and metabolic dysfunction in Malaysian Malay pregnant women with GDM.

It is important to note that the observed microbial variations may not solely be a cause but rather a consequence or marker of GDM. Further research is needed to establish causality and elucidate the complex interplay between gut microbiota, metabolic processes, and GDM development in Malaysian Malay pregnant women with GDM.

### Study’s strengths

Overall, the study design and methodology possess several strengths that contribute to the quality and credibility of the findings. The prospective cohort design, inclusion of control and condition groups, comprehensive assessment of variables, utilization of advanced molecular techniques, and use of validated measurement tools collectively enhance the scientific rigor of the study. These strengths increase the reliability and generalizability of the findings, making them valuable contributions to the existing knowledge on the gut microbiome, GDM, and associated factors during pregnancy.

### Study’s limitations and potential sources of bias

While this study has provided valuable insights into the gut microbiome to GDM and pregnancy outcomes, it also acknowledged and explored the limitations and potential sources of bias that may have influenced the results. One potential limitation is the relatively small sample size of the study. The sample size in both the GDM and non-GDM groups may limit the generalizability of the findings to larger populations. A larger sample size would allow for more robust statistical analyses and uncover additional associations. Furthermore, the study was conducted at only two clinics in the southern part of Malaysia, which may introduce some site-specific biases and limit the generalizability of the findings to another setting or population within Malaysia and worldwide. Secondly, the study relied on self-reported measures for various variables, including dietary intake, physical activity, and lifestyle factors. Self-reported data are subject to recall bias and social desirability bias, which may impact the accuracy and reliability of the information collected. Thirdly, the study's findings are based on associations and correlations rather than mechanistic explanations. The underlying mechanisms remain unclear, but the associations between the gut microbiome and various factors have been identified.

## Conclusion

In conclusion, the study delved into the nuanced landscape of GDM among Malaysian Malay women, revealing significant disparities in clinical factors, dietary patterns, and lifestyle habits during pregnancy. Participants with GDM exhibited elevated glucose levels, increased body weight, and distinct lifestyle variations. The exploration of gut microbial profiles highlighted a dynamic shift in diversity, particularly a reduction in alpha diversity in the GDM group during the third trimester. The findings highlight the intricate interplay between GDM, maternal health parameters, and the evolving gut microbiome.

### Recommendations

Future research should focus on elucidating the underlying mechanisms, exploring longitudinal changes, investigating the impact of dietary interventions, studying the gut-brain axis, considering the interplay with host genetics, and examining environmental factors in the context of GDM and the gut microbiome. Addressing these knowledge gaps will advance our understanding of the complex interactions between the gut microbiome, GDM, and maternal and neonatal outcomes and pave the way for targeted interventions and improved clinical management strategies for Malay Malaysian pregnant women.

### Supplementary Information

Supplementary Table 1.

Supplementary Table 2.

Supplementary Table 3.

Supplementary Table 4.

## Data Availability

The datasets generated and analyzed during the study will not be publicly available due to patient confidentiality rules.
