# Supplementary material for: Diet, lifestyle and gut microbiota composition among Malaysian women with gestational diabetes mellitus: a prospective cohort study
Source: Sci Rep. 2024 Mar 22;14:6891. doi: 10.1038/s41598-024-57627-5 (PMC10959929; doi:10.1038/s41598-024-57627-5)
Supplement: Supplementary file 1 — Supplementary Table 1. [file 41598_2024_57627_MOESM1_ESM.docx]

| **Section** | **Description** | **Timepoint** | |
| --- | --- | --- | --- |
|  |  | **T0** | **T1** |
| 1. Demography | Date of birth (age), education level, marital status, occupation, and personal and household income. | ✓ |  |
| 2. Clinical | Clinical characteristics (history of GDM, family history of diabetes, blood pressure, pre-pregnancy weight, height, weight at enrollment and third trimester) and biomarkers (OGTT at enrolment, fasting blood glucose)  were obtained from medical records. | ✓ | ✓ |
| 3. Lifestyle | Lifestyle habits (Smoking, alcohol, sleeping, and exercise habits)  Global Physical Activity Questionnaire (GPAQ) (43) | ✓ | ✓ |
| 4. Diet | Three-day 24-hour dietary recalls, supplementary intake | ✓ | ✓ |

**Supplementary Table 1**: Study instrument
