# Supplementary material for: Diet, lifestyle and gut microbiota composition among Malaysian women with gestational diabetes mellitus: a prospective cohort study
Source: Sci Rep. 2024 Mar 22;14:6891. doi: 10.1038/s41598-024-57627-5 (PMC10959929; doi:10.1038/s41598-024-57627-5)
Supplement: Supplementary file 2 — Supplementary Table 2. [file 41598_2024_57627_MOESM2_ESM.docx]

**Supplementary Table 2:** Enrichment of specific microbial taxa in two groups (non-GDM and GDM) at T0 and T1.

| **Taxa** | **Enriched_group** | **ef_lda** | **p-value** | **FDR** |
| --- | --- | --- | --- | --- |
| *Oscillospira* | non-GDM_T0 | 2.624 | 0.018 | 0.018 |
| *Elusimicrobium* | non-GDM_T0 | 2.515 | 0.026 | 0.026 |
| *Terrisporobacter* | non-GDM_T0 | 2.093 | 0.010 | 0.010 |
| Ruminococcaceae | non-GDM_T1 | 3.840 | 0.049 | 0.049 |
| *Bacteroides_caccae* | non-GDM_T1 | 3.555 | 0.006 | 0.006 |
| *Roseburia* | non-GDM_T1 | 3.388 | 0.003 | 0.003 |
| *Wadsworthia* | non-GDM_T1 | 3.382 | 0.004 | 0.004 |
| *Oscillibacter* | non-GDM_T1 | 3.323 | 0.005 | 0.005 |
| *Incertae Sedis* | non-GDM_T1 | 3.006 | 0.013 | 0.013 |
| *Eggerthellaceae* | non-GDM_T1 | 2.758 | 0.007 | 0.007 |
| *Lachnospiraceae UCG010* | non-GDM_T1 | 2.758 | 0.019 | 0.019 |
| *UBA1819* | non-GDM_T1 | 2.643 | 0.011 | 0.011 |
| *Flavonifractor* | non-GDM_T1 | 2.450 | 0.024 | 0.024 |
| *[Clostridium] innocuum* group | non-GDM_T1 | 2.326 | 0.044 | 0.044 |
| *Butyriciproducens* | non-GDM_T1 | 2.323 | 0.011 | 0.011 |
| *Raoultibacter_timonensis* | non-GDM_T1 | 2.082 | 0.002 | 0.002 |
| *Phocea_massiliensis* | non-GDM_T1 | 2.021 | 0.027 | 0.027 |
| *[Eubacterium] fissicatena group* | non-GDM_T1 | 2.009 | 0.007 | 0.007 |
| *DTU089* | non-GDM_T1 | 2.003 | 0.001 | 0.001 |
| *Ovatus* | GDM_T0 | 3.038 | 0.005 | 0.005 |
| *Shahii* | GDM_T1 | 2.900 | 0.002 | 0.002 |

**Note:** ef_lda: The linear discriminant analysis (LDA) estimated the effect size. It measures the magnitude of the difference in abundance between the groups. Higher values indicate a greater difference in abundance. p-value; The p-value associated with the enrichment of the taxa in the specified group. It indicates the statistical significance of the enrichment. FDR: The false discovery rate (FDR) associated with the enrichment of the taxa. FDR is a multiple-testing correction method that controls the proportion of false positives among the rejected hypotheses.
