# Supplementary material for: Diet, lifestyle and gut microbiota composition among Malaysian women with gestational diabetes mellitus: a prospective cohort study
Source: Sci Rep. 2024 Mar 22;14:6891. doi: 10.1038/s41598-024-57627-5 (PMC10959929; doi:10.1038/s41598-024-57627-5)
Supplement: Supplementary file 3 — Supplementary Table 3. [file 41598_2024_57627_MOESM3_ESM.docx]

**Supplementary Table 3:** Correlation of genus-level gut microbial abundance with detected predictors in the GDM group in the second trimester.

| **Genus** | **Predictors** | **Correlation** | **Adjusted p-value** |
| --- | --- | --- | --- |
| *Lactiplantibacillus* | Physical activity level | 1.00 | <0.001** |
| *Parvibacter* | Physical activity level | 1.00 | <0.001** |
| *Prevotellaceae UCG001* | Physical activity level | 1.00 | <0.001** |
| *Vagococcus* | Physical activity level | 1.00 | <0.001** |
| *Victivallis* | Gravida | 0.90 | 0.003* |
| *Victivallis* | Parity | 0.90 | 0.003* |

*Significant at p<0.005; **significant at p<0.001.
