# Supplementary material for: Diet, lifestyle and gut microbiota composition among Malaysian women with gestational diabetes mellitus: a prospective cohort study
Source: Sci Rep. 2024 Mar 22;14:6891. doi: 10.1038/s41598-024-57627-5 (PMC10959929; doi:10.1038/s41598-024-57627-5)
Supplement: Supplementary file 4 — Supplementary Table 4. [file 41598_2024_57627_MOESM4_ESM.docx]

**Supplementary Table 4:** Correlation of genus-level gut microbial abundance with detected predictors in the non-GDM group at T1.

| **Genus** | **Predictors** | **Correlation** | **Adjusted p-value** |
| --- | --- | --- | --- |
| *Bacteroides* | omega-3 PUFAs | -0.93 | 0.003** |
| *Roseburia* | omega-3 PUFAs | 0.90 | 0.014* |

*Significant at p<0.05**significant at p<0.005; Omega3 PUFA refers to Omega-3 polyunsaturated fatty acids intake per 1000 kcal.
